# *Ephedra sinica* Stapf and Gypsum Attenuates Heat-Induced Hypothalamic Inflammation in Mice

**DOI:** 10.3390/toxins12010016

**Published:** 2019-12-30

**Authors:** Wonnam Kim, Wonil Lee, Eugene Huh, Eunjung Choi, Young Pyo Jang, Yun-Kyung Kim, Tae-Hee Lee, Myung Sook Oh

**Affiliations:** 1 Division of Pharmacology, College of Korean Medicine, Semyung University, 65 Semyung-ro, Jecheon 27136, Korea; 2 Department of Life and Nanopharmaceutical Sciences, Graduate School, Kyung Hee University, 26 Kyungheedae-ro, Dongdaemun-gu, Seoul 02447, Korea; 3 Department of Medical Science of Meridian, Graduate School, Kyung Hee University, 26 Kyungheedae-ro, Dongdaemun-gu, Seoul 02447, Korea; 4 Department of Oriental Pharmaceutical Science, College of Pharmacy Kyung Hee University, 26 Kyungheedae-ro, Dongdaemun-gu, Seoul 02447, Korea; 5 Department of Herbal Medicine, College of Pharmacy, Wonkwang University, 460 Iksan-daero, Iksan 54538, Korea; 6 Department of Formulae Pharmacology, School of Oriental Medicine, Gachon University, 1342 Seongnamdae-ro, Sujeong-gu, Seongnam 13120, Korea; 7 Kyung Hee East-West Pharmaceutical Research Institute, Kyung Hee University, 26 Kyungheedae-ro, Dongdaemun-gu, Seoul 02447, Korea

**Keywords:** *Ephedra sinica* Stapf, gypsum, heat stress, hypothalamus, inflammation

## Abstract

*Ephedra sinica* Stapf (EH) exert toxic effects, such as excitability, cardiac arrhythmia, and others. On the contrary, in traditional herbal medicine, EH and gypsum (GF) are used most often to treat symptoms caused by external stressors. The hypothalamus plays a crucial role in thermal homeostasis. Inflammatory response in the hypothalamus by thermal stressors may affect thermal and energy homeostasis. This study investigates the effect of EH and GF against heat-induced mouse model. Mice were divided into four groups: saline, saline plus heat, EH plus heat, and GF plus heat treated groups. Heat stress was fixed at 43 °C for 15 min once daily for 3 days. Weight and ear and rectal temperature measurements were made after terminating heat stress. Hypothalamus tissue was collected to evaluate the HSP70, nuclear factor kappa-Β (NF-kB), and interleukin (IL)-1β protein expression levels. EH and GF treatment suppressed the increased body temperature. EH significantly ameliorated heat-induced body weight loss, compared to gypsum. Regulatory effects of EH and GF for body temperature and weight against heat stress were mediated by IL-1β reduction. EH showed significant HSP70 and NF-kB inhibition against heat stress. EH and GF contribute to the inhibition of heat-induced proinflammatory factors and the promotion of hypothalamic homeostasis.

## 1. Introduction

Traditionally, the dried stems and leaves of *Ephedra sinica* Stapf (EH) were frequently used to treat symptoms such as cough, wheezing, and asthma [1]. However, the prescribing and monitoring of EH requires caution because excessive dose or prolonged duration are more likely to cause adverse effects [2]. The reported toxic effects of EH are tremor, excitability, headache, anxiety, insomnia, nausea, vomiting, poor appetite, cardiac arrhythmia, convulsions, and others [3]. Despite the toxicity of EH, EH in combination with gypsum (GF) is the most common herbal treatment for fever and asthma [4]. Mahaenggamseok-tang (Ma-Xing-Shi-Gan-Tang in Chinese, Makyokansekito in Japanese), a herbal formula, is frequently used in Korea, China, and Japan to improve symptoms related to upper respiratory infection, particularly cough and fever [5,6]. Based on traditional Asian medicine, Mahaenggamseok-tang was formulated to manage disorders by external stressors, such as temperature variations. Mahaenggamseok-tang is composed of EH, *Prunus armeniaca* Linne var. ansu Maximowicz, *Glycyrrhiza uralensis* Fischer, and GF [7]. Among these herbal compositions EH and GF are the main components of Mahaenggamseok-tang that act to regulate the body temperature affected by thermal stress.

Thermal homeostasis is characterized as the maintenance of a relatively stable body temperature within a certain range (33.2–38.2 °C) [8]. Deviation from the normal body temperature will cause thermoregulatory challenges, and temperature values outside the reference range can potentially be life-threatening [8]. The hypothalamus is the thermoregulatory center responsible for integrating and coordinating various kinds of thermal information [9]. The anterior hypothalamus-preoptic area is the most important region involved with autonomic thermoregulatory mechanisms [10]. Our previous work has shown that in response to thermal stressors, hypothalamic inflammation is linked to proinflammatory factors, such as IL-9 and IL-13 [11,12]. Inflammation of the hypothalamus leads to complications such as metabolic syndrome, which manifests a diverse clinical spectrum from obesity to cachexia. [13].

Little is known about the thermoregulatory functions regarding the broad clinical use of EH and GF. This study aimed to explore the thermoregulatory effect of EH and GF against heat stress. We further investigated the contribution of EH and GF to the inhibition of hypothalamic inflammation.

## 2. Results

### 2.1. Identification of Ephedra sinica Stapf Extract (EHE) and Gypsum

**Figure 1:**
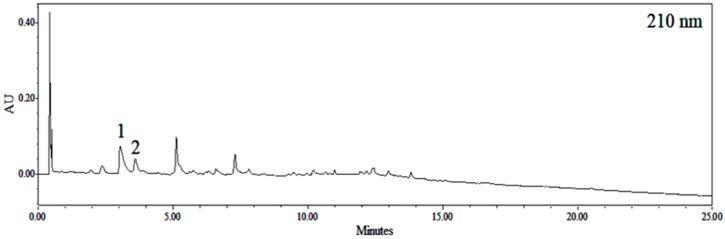
Ultra-performance liquid chromatography (UPLC) chromatogram of *Ephedra sinica* Stapf extract detected at 210 nm. 1 Ephedrine, 2 Pseudoephedrine.

**Figure 2:**
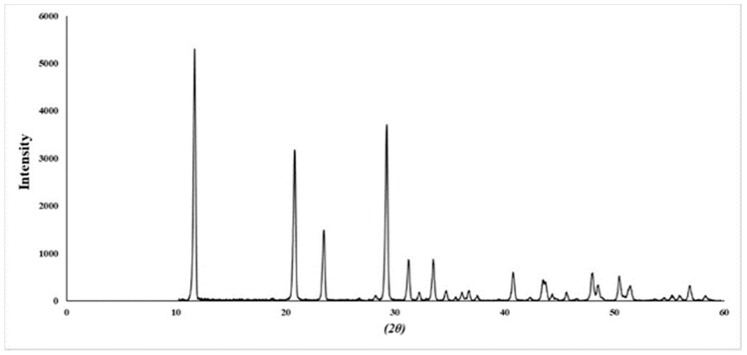
XRD spectra of gypsum.

The ultra-performance liquid chromatography (UPLC) chromatogram of EHE detected at 210 nm (Figure 1). Two major peaks were identified by ultra-performance liquid chromatography-photodiode array detector-electrospray-mass spectrometry (UPLC-PDA-ESI-MS), the list of retention times, precursor ions, and molar masses of each compound are shown in Table 1. Peak 1 was confirmed as ephedrine, since it had a *m*/*z* value of 166.11484, which gave a mass difference of −8.4 mmu compared to the theoretical value of ephedrine and retention order in reversed phase silica-based chromatography. Peak 2 was confirmed as pseudoephedrine by direct comparison of its spectroscopic and chromatographic characteristics with the data previously reported [14]. The X-ray diffraction (XRD) of GF is shown in Figure 2. The diffraction data typically provided the same pattern as calcium sulfate dihydrate [15].

### 2.2. Effects of Ephedra sinica Stapf Extract and Gypsum Extract (GFE) on Heat-Induced Changes in the Body Temperature and Weight of Mice

**Figure 3:**
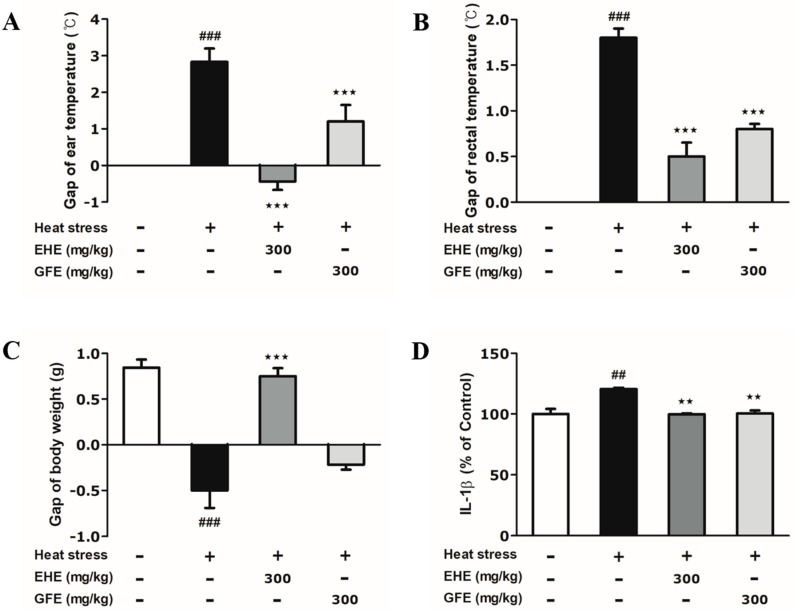
Effects of *Ephedra sinica* Stapf extract (EHE) and gypsum extract (GFE) on heat-induced changes in body temperature, weight and IL-1β levels. (**A**,**B**) Ear and rectal temperature changes represent the difference between day 0 and 3. (**C**) Body weight change represents the difference between day 0 and 3. (**D**) IL-1β levels measured from hypothalamic lysates. Data expressed as mean values (±SEM) (**A**–**C**: *N* = 4; **D**: *N* = 3). + Stimulation, − Non-stimulation ^#^ Refers to significant difference with the control group (^##^ *p* < 0.01, ^###^ *p* < 0.001). ^★^ Refers to significant difference with the heat-only group (^★★^ *p* < 0.01, ^★★★^ *p* < 0.001).

Heat stress induced significant changes in body temperature and weight measurement compared to control group (Figure 3A–C). The EHE-treated (ear: −0.43 °C; rectal: 0.5 °C) and GFE-treated (ear: 1.3 °C; rectal: 0.8 °C) groups significantly suppressed elevated ear and rectal temperature by heat stress (ear: 2.8 °C; rectal: 1.8 °C) (Figure 3A,B). The EHE-treated group (0.75 g) significantly increased body weight compared to heat-only group (−0.5 g) (Figure 3C). However, the increase in body weight in the GFE-treated group was not significant. Based on these data, EHE and GFE treatment altered changes in body temperature and weight induced by heat stress.

### 2.3. Effects of EHE and GFE on Heat-Induced IL-1β Changes in the Hypothalamus

Based on the effective regulation of body temperature and weight by EHE and GFE under heat stress, we next determined the heat-induced inflammatory cytokines in the hypothalamus. As IL-1β is an emerging therapeutic target for inflammatory conditions, we evaluated the changes of IL-1β levels [16,17]. Heat stress caused a 1.2-fold increase in IL-1β expression compared to the non-stimulated control group (Figure 3D). Treatment with EHE and GFE each significantly decreased IL-1β levels by 0.8-fold compared to the heat-only group (Figure 3D). The data indicates that both EHE and GFE suppress the overexpression of IL-1β in the hypothalamus by heat stress.

### 2.4. Effects of EHE and GFE on Heat-Induced Biochemical Changes in the Hypothalamus

**Figure 4:**
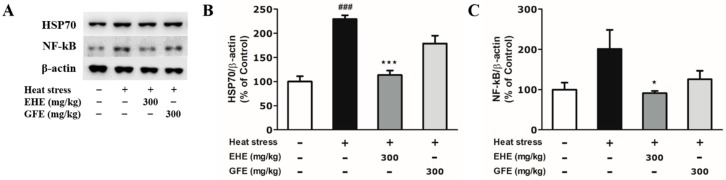
Effects of *Ephedra sinica* Stapf extract (EHE) and gypsum extract (GFE) on heat-induced biochemical changes in the hypothalamus. (**A**) Western blot analysis with HSP70, NF-kB, and β-actin from hypothalamic lysates. (**B**,**C**) Quantitative data of HSP70 and NF-kB expression levels. Data expressed as mean values (±SEM) (*N* = 3). + Stimulation, − Non-stimulation. ^#^ Refers to significant difference with the control group (^###^ *p* < 0.001). ^★^ Refers to significant difference with the heat-only group (^★^ *p* < 0.05, ^★★★^ *p* < 0.001).

To determine the thermal stress-related biological response in the hypothalamus, we examined the HSP70 and NF-kB protein expression. Mice exposed to heat stress tended to show increased HSP70 and NF-kB expression (Figure 4A–C). Our data are in parallel with previous studies that have showed that heat stress increases HSP70 and NF-kB levels in the hypothalamus [11,17]. The HSP70 expression of EHE-treated mice was significantly downregulated (by 0.5-fold) compared to the heat-only group (Figure 4B). The NF-kB expression of EHE-treated mice was significantly downregulated (by 0.5-fold) compared to the heat-only group (Figure 4C). However, suppression of HSP70 and NF-kB levels was not significant in the GFE-treated group (Figure 4B,C). These data indicate that EHE inhibits biological change in the hypothalamus related to heat-induced thermal stress.

## 3. Discussion

In traditional herbal medicine, Mahaenggamseok-tang has been used to regulate increased core body temperature when a patient is presenting with respiratory infection [18]. For the first time, we demonstrated that EH and (to a lesser degree) GF, the main components of Mahaenggamseok-tang, might lead to thermal homeostasis during heat stress in the hypothalamus. Given that Mahaenggamseok-tang aids in cooling down the temperature increase, we were interested in determining whether EHE and GFE might mediate these effects. We found that both EHE and GFE mitigated the increase in body temperature resulting from heat stress. In order to understand the mechanism of thermoregulation by EHE and GFE against heat stress, we determined the hypothalamic IL-1β levels. The proinflammatory cytokines IL-1β, IL-6, and tumour necrosis factor (TNF)-α are synthesized in the hypothalamus by lipopolysaccharide (LPS) administration [19,20]. In the hypothalamus, IL-1β is reported to be responsible for the LPS-mediated anorexia and secretion of proinflammatory cytokines [21]. EHE and GFE significantly attenuated heat-induced hypothalamic IL-1β overexpression. This modification in IL-1β levels could, in part, explain the weight gain by the EHE and GFE-treated group compared to the heat-only group. Numerous animal studies have shown that food consumption decreases as environmental temperatures increase in order to affect homeostasis [22]. GFE treatment showed a tendency to inhibit the body weight reduction by heat stress, but this tendency was not significant. However, EHE treatment significantly increased body weight compared to the heat-induced weight loss. This is an interesting observation, considering that EH is widely consumed in weight loss products in humans [23]. In mice, high fat diet (HFD) tends to result in gains in body weight, but a HFD consisting of 5% EH has been shown to result in a loss of body weight [24]. Additionally, intracerebroventricular administration of IL-1β increased rectal temperature in rabbits [25]. In parallel, our results demonstrate that heat stress induced an increase in rectal temperature and IL-1β level. Therefore, the underlying mechanism inhibiting temperature increase by EFE and GFE can be explained by IL-1β. NF-kB is an important transcription factor for proinflammatory cytokines, including IL-1β and TNF-α [26]. Our data support that the inhibitory effects of EHE in IL-1β expression are mediated by NF-kB. A previous study has shown that EH inhibits NF-kB activation induced by lipopolysaccharide stimulation in rat glioma cell line [27]. It is unclear how to interpret the IL-1β reduction by GFE, especially when NF-kB suppression is not significant. Additional research is needed to understand the overall process.

The hypothalamus plays a significant part in homeostasis against stressors. We found that EHE and GFE ameliorates the proinflammatory response in the hypothalamus resulting from heat stress. The recovery of body weight and body temperature by EHE and GFE against heat stress is mediated through IL-1β. In that case, EH and GF may offer a hypothalamic thermoregulatory effect and energy balance under heat stress. Our study provides new evidence to help understand the molecular basis for the traditional use of EH and GF. Moreover, heat stress has a considerable impact on health and performance of humans and also livestock. We hope that our study will help in providing new insights for treatments aimed not only at ameliorating the adverse effects of heat stress but also at building resilience against heat stress.

## 4. Materials and Methods

### 4.1. Materials

Acetonitrile, for HPLC, was purchased from Thermo Scientific (Waltham, MA, USA) and trifluoroacetic acid (HPLC-grade) was obtained from Sigma Aldrich (Burlington, MA, USA). HSP70 (goat Ab), NF-kB (rabbit Ab), and β-actin (mouse Ab) were supplied by Santa Cruz (Santa Cruz Biotechnology, Inc., Dallas, TX, USA). Horseradish peroxidase (HRP) secondary antibodies were from Enzo Life Sciences (Nassau, NY, USA). PVDFs were obtained from Millipore (Burlington, MA, USA). Mouse anti-IL-1β ELISA kit was purchased from Ray Biotech (Norcross, GA, USA). Sodium chloride, ethanol, and bovine serum albumin were from Sigma Aldrich. Bradford assay, Tween 20, ammonium persulfate, acrylamide, chemiluminescent substrate reagent, and nonfat dry milk were supplied from Bio-Rad (Hercules, CA, USA).

### 4.2. Preparation of EH and GF

Dried EH and GF were obtained from Jungdo Herb Co. (Seoul, Korea). EH and GF were boiled in 70% ethanol (2 L) and filtered over Whatman #1 paper (GE Healthcare, Chicago, IL, USA). The filtrate was evaporated under reduced pressure on a rotary evaporator. Dry extracts were successively obtained from a yield of 17.28% (EH) and 2.18% (GF). The lyophilized powders were dissolved with distilled water, and saline dilution was done before treatment by oral gavage.

### 4.3. UPLC-PDA-ESI-MS Identification

AcquityTM ultra-performance liquid chromatography (UPLC) H-class system (Waters Co., Milford, MA, USA) analysis was performed using a photo diode array (PDA) detector (Waters Co., Milford, MA, USA). Electrospray ionization mass spectra (ESI-MS) were performed with a JMS-T100TD spectrometer (JEOL Ltd., Tokyo, Japan). Kinetex EVO C18 column (100 × 2.1 mm, 1.7 µm, 100 Å; Phenomenex, Torrance, CA, USA) was used for chromatographic separation. Column oven temperature was set at 40 °C, with an injection volume of 1.0 µL, and flow rate of 0.5 mL/min. The mobile phase consisted of solvent A (acetonitrile) and solvent B (0.1% trifluoroacetic acid). The elution conditions applied were as follows: 0–16 min, 0% to 16%; 16–20 min, 16% to 100%; 20–25 min, 100% solvent A. Ion source settings were as follows: scan range, 50–1000 *m*/*z*; N_2_ gas, nebulizing gas flow rate, 1.0 L/min; desolvation gas flow rate, 3.0 L/min; desolvating chamber temperature, 250 °C; orifice1 temperature, 80 °C; ring lens voltage, 5 V; orifice 1 voltage, 80 V; orifice 2 voltage, 10 V; detector voltage, 1900 V; and peak voltage, 1000 V.

### 4.4. XRD Analysis

X-ray diffraction (XRD) was performed using DMAX-IIIA (Rigaku, Akishima-shi, Japan) with 2 kW X-ray source. Well-grounded GF samples were positioned on a silicon wafer, and data collection was done between 10° and 60°. The composition of the precipitates was determined using an ATSAS 2.7 (BIOSAXS GmbH, Hamburg, Germany).

### 4.5. Animals and Measurement

Seven-week-old male Institute of Cancer Research (ICR) mice (30–32 g) were obtained from Orient Co. (Seoul, Korea). Mice were maintained 10 per cage (40 × 25 × 18 cm) with adequate access to food and water, in a 12-h dark:light cycle at a temperature of 23 ± 1 °C and humidity of 60 ± 10%. On arrival, the mice were acclimatized for 1 week before the experiments started. Mice were randomly divided into four groups: group 1 (saline; *N* = 7), group 2 (saline; *N* = 7), group 3 (EHE 300 mg/kg/day; *N* = 8), group 4 (GFE 300 mg/kg/day; *N* = 8). Groups 2–4 were exposed to heat stress. On each of the three testing days, mice were transferred to a heat exposure chamber (Jeiotech Co., Seoul, Korea) fixed at temperature of 43 °C and humidity of 60 ± 10% for 15 min/day [11]. To prevent the influence of diurnal cycling, the time was kept constant for heat exposure each day. Weight was measured on an electronic scale (Ohaus Co., Shanghai, China). Temperature was measured by inserting a probe inside the ear and rectum (TC-100, CWE Inc., Ardmore, PA, USA). After thermal exposure ended, mice were returned to their housing cage. All animal experimental procedures were in accordance with the guidelines of Animal Care and Use, Kyung Hee University (identification number: KHP-2014-05-3; approval date: 30 May 2014).

### 4.6. Western Blot Analysis

Mice were sacrificed 2 h after the last exposure to heat. The hypothalamic tissues were immediately frozen and kept at −80 °C until use. Samples were separated on a 10% SDS-PAGE and then transferred to a PVDF membrane. Blocking was done with 5% nonfat milk in Tween 20/Tris-buffered saline for 1 h. Overnight incubation occurred at 4 °C with primary antibodies (1:1000) and then 1 h of incubation occurred at room temperature (RT) with HRP-conjugated secondary antibody. The immuno-detection bands were developed with an ECL agent and visualized with a LAS-4000 Mini system. Quantification was done by densitometric analysis (Multi-gauge, Fujifilm Co., Tokyo, Japan).

### 4.7. IL-1β Expression Analysis

Mouse IL-1β was quantified using ELISA assay kit according to the manufacturer’s instructions. In brief, incubation of lysate and reaction buffer mix occurred for 2.5 h in RT and was followed by measurement with a microplate reader (excitation: 360 nm and emission: 450 nm).

### 4.8. Statistical Analysis

All calculations for statistical analysis were evaluated with GraphPad Prism (version 5.0, GraphPad Software, San Diego, CA, USA). Results are expressed as means ± SEM. ANOVA with Tukey’s post hoc test was used. In this study, *p* values of <0.05 were regarded as statistically significant.

## Figures and Tables

**Table 1:** Peak identification of *Ephedra sinica* Stapf by ultra-performance liquid chromatography-photodiode array detector-electrospray-mass spectrometry (UPLC-PDA-ESI-MS).

Compound	Rt (min)	Precursor ion (*m*/*z*)	Molar Mass (g/mol)	λ max (nm)
1. Ephedrine	2.84	166.11484 [M+H]^+^ 148.10664 [M−H_2_O+H]^+^ 133.08700 [M−H_2_O−CH_3_+H]^+^	165.115	206
2. Pseudoephedrine	3.38	166.11544 [M+H]^+^ 148.10682 [M−H_2_O+H]^+^ 133.08706 [M−H_2_O−CH_3_+H]^+^	165.115	206
